# Surgical simulator for temporal bone dissection training

**DOI:** 10.1590/S1808-86942010000500007

**Published:** 2015-10-22

**Authors:** Daniel Mochida Okada, Ana Maria Almeida de Sousa, Raul de Andrade Huertas, Fabio Akira Suzuki

**Affiliations:** 1 Clinical and surgical otology expert - UNIFESP, Assistant Physician - Otolaryngology Department - Hospital do Servidor Público Estadual - IAMSPE; 2 ENT Resident - Hospital do Servidor Público Estadual - IAMSPE; 3 Dental prosthesis technician - Colégio Bernardino de Campos, Sócio-proprietário J&R Laboratório de Próteses Dentárias; 4 PhD in Otolaryngology - UNIFESP, Coordinator of the Internship - Hospital do Servidor Público Estadual - IAMSPE. Hospital do Servidor Público Estadual - Instituto de Assistência Médica ao Servidor Público Estadual Serviço de Otorrinolaringologia - Diretor Dr. Samir Cahali

**Keywords:** surgery, dissection, temporal bone.

## Abstract

**Abstract:**

Temporal bone dissection plays an important role in the training of surgeons; however, they are difficult to obtain.

**Aim:**

To develop a synthetic replica of the temporal bone for dissection training.

**Study Design:**

Experimental.

**Materials and Methods:**

An acrylic synthetic resin replica was obtained from a human temporal bone. For the evaluation of the method, we selected five ear surgeons to dissect the model in a laboratory of experimental surgery. A questionnaire was filled, assessing external appearance, the simulation of procedures (placement of ventilation tube, mastoidectomy, decompression of the facial nerve and translabyrinthine access to the internal auditory canal) and their final conclusion.

**Results:**

The evaluation indicated satisfaction in using the model (80%), being more evident concerning the dissection of the mastoid segment of the facial nerve and translabyrinthine access to the internal auditory canal. The placement of a ventilation tube was reasonable for 60% and satisfactory for 40% of them. Mastoidectomy was satisfactory for 60% and fully satisfactory for 40%.

**Conclusion:**

Dissection in this simulator does not replace otologic training in cadaveric temporal bones. However, given the increasing difficulty in obtaining the latter, the development of new teaching tools should be encouraged to continuously improve surgeons.

## INTRODUCTION

The use of cadavers for medical education and training started in 500 B.C. and its history evolved hand-in-hand with the development we have today in Medicine. For many years, this practice was prohibited because of religious and ethical issues; however, it was after the renascence that Leonardo da Vinci, Michelangelo and Andreas Vesalius consolidated human anatomy and dissection methods^1^.

Study with cadavers is fundamental in schools of medicine, nursing, physical therapy and other health-care disciplines. Training of surgical techniques in cadavers also plays an important role in the education and enhancement of surgeons. The current Brazilian legislation, by means of law # 8,501 from November 30 of 1992, establishes that only those cadavers which remain unclaimed for 30 days can be used for research and educational purposes^2^.

Training in temporal bone dissection laboratories is already routinely carried out for the learning of surgical anatomy and to train otolaryngologists, in accordance with the theories from Fitts and Posner for the training of motor skills^3^.

Thus, if on the one hand we have the legislative and the difficulty in obtaining cadavers representing a major limitation, on the other we have the need to develop new teaching approaches such as simulators and training in virtual environments.

The goal of this project was to develop a replica of the temporal bone, with proper anatomical reliance in order to train dissection in the laboratory.

## MATERIALS AND METHODS

The project was analyzed and approved by the Ethics in Research Committee, through protocol # 054/09.

As primary model we used a left side temporal bone we acquired from the Coroner's Office of the city of São Paulo, through an official request # 18/2008.

**Figure 1:**
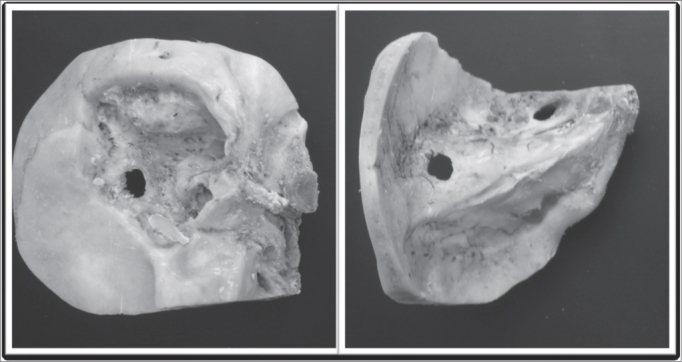
Human temporal bone after broad mastoidectomy.

After properly cleaning the specimen, we did a broad mastoidectomy in order to identify the sigmoid sinus, mastoid tegmen, vertical portion of the facial nerve and lateral semi-circular canal. After drilling the posterior wall of the auditory meatus we removed the tympanic membrane and ossicular chain. (Fig. 1)

**Figure 2:**
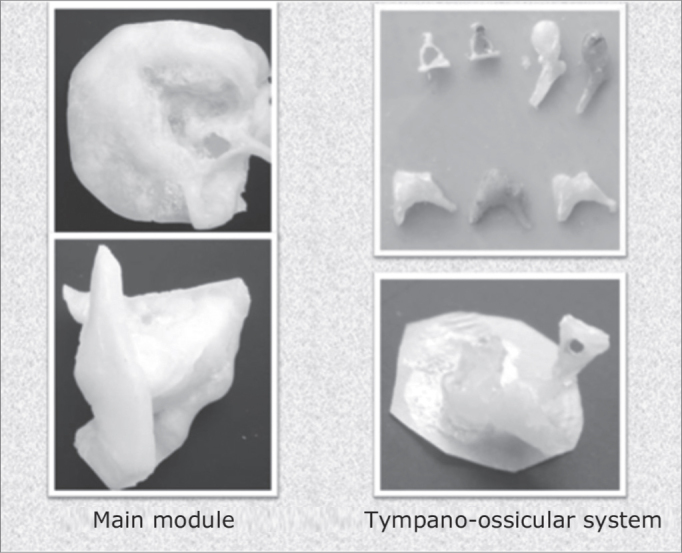
Main module and tympano-ossicular system.

**Figure 3:**
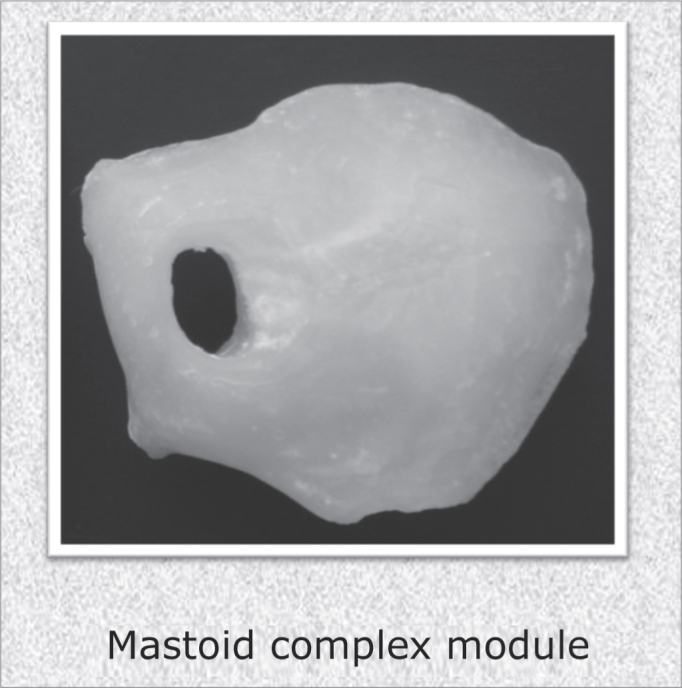
Mastoid complex module.

Using molding, high fidelity silicone and thermocuring acrylic (Reg. ANVISA 10234680006) we built three modules (main, tympano-ossicular system and mastoid complex) for later assemblage. The tympanic membrane was built from vegetable parchment paper. We stress that the technique used to build the mastoid complex was changed in order to obtain a porous material, similar to the pneumatization of the temporal bone region. (Figs. 2 and 3)

**Figure 4:**
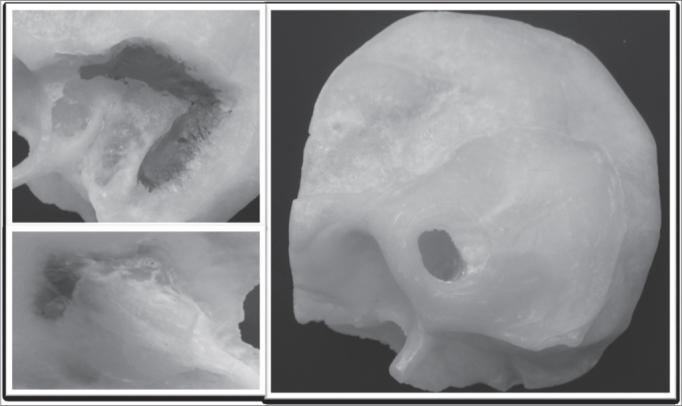
Surgical simulator.

Before assembling the three modules we painted some of the structures with acrylic paint, namely: (Figure 4)

– Blue: sigmoid sinus
– Red: mastoid tegmen
– Yellow: mastoid portion of the facial nerve and internal acoustic meatus

In order to evaluate the simulator we selected five experienced ear surgeons skilled in experimental dissection to study the models and later fill out the questionnaire, taking into account the external appearance, the simulation of procedures (ventilation tube insertion, mastoidectomy, decompression of the mastoid portion of the facial nerve and translabyrinthine access to the internal acoustic meatus) and their final conclusion. The aforementioned procedures were done in an experimental surgery lab, with the equipment routinely used to dissect temporal bones.

## RESULTS

The opinions of the surgeons are listed on the following table. Each mark (*) represents the opinion of each surgeon. (Table 1)

**Table 1:** 

	Identical	Very similar, but not identical	More or less alike	Very different	Totally different
External appearance	*	****			
	Totally satisfactory	Satisfactory	Reasonable	Bad	Very bad
Ventilation tube insertion		**	***		
Mastoidectomy	**	***			
Dissection of the mastoid segment of the facial nerve	****		*		
Translabyrinthine access to the internal acoustic meatus					
	Completely satisfied	Satisfied	Reasonable	Bad	Very bad
Final opinion	*	****			

## DISCUSSION

Before 1992 there were no laws regulating the use of cadavers for educational purposes. Different problems were faced by medical schools in attempting to find bodies from indigents^4^. The already known ethical and religious issues blocked the donation of unidentified cadavers^5^. Financial aspects, such as the provision of funeral grant by Social Security also made this process even more difficult^5^.

Law # 8.501 from November 30, 1992 rules the conditions of cadaver destination for education and research. The cadavers which are unidentified and unclaimed after thirty days can then be sent to educational institutions. Should the cadaver be identified; however, without information from relatives or legal guardians, local authorities should publish the information in the main newspapers of the city during at least ten days, as a public service. Bodies suspected of unnatural or criminal cause of death cannot be used. Moreover, the law establishes that for recognition purposes, the authority or institution in charge shall keep the following information regarding the deceased: general characteristics, photos, fingerprints and identification - should there be any^2^.

Nonetheless, knowing that the cadaver is only donated after thirty days and that often times it is not put into formaldehyde before 72 hours post-mortem, the degeneration process makes its use impossible6. Moreover, we have seen the spread of medical and other health care disciplines schools, which increases even further the demand for cadavers.

These factors, associated with the constant pressure for better results have led educators and researchers to develop new tools to train surgeons^7^. Thus, synthetic simulators and those based on virtual environments are being developed in order to make up for the lack of cadavers^8^,^9^.

Fitts and Posner's theory shows that learning is aninternal human process, and it depends on practice and it tends to be permanent. Such process happens in three stages - cognitive, associative and autonomous - in which, as the individual progresses on his/her training, better are the outcomes achieved, its consistency and less number of mistakes, together with an increase in the person's capacity to identify and correct problems^3^.

Training in simulators, as well as conventional dissection, can separate the acquisition of skills from medical practice^10^. In the field of otology, there is already a virtual simulator of temporal bone dissection (VOXEL-MAN Temposurg Simulator) which enables the three-dimensional visualization of tissues and provides a sensitive answer to the operator (resistance, directions, burr speed). Zirkle et al.^11^ believe that such equipment has the potential to enhance the skills of ENT residents. Nonetheless, this equipment does not allow the use of routine equipment used in otologic surgery, such as a microscope, aspirator and micromotor. Moreover, its high cost makes it difficult to spread its use.

The present study aims at showing that this simulator enables temporal bone dissection training in conventional labs, for it bears proper anatomical reliance and provides a realistic feeling during surgery. Nonetheless, some problems have been raised, such as tympanic membrane stiffness, the greater resin density when compared to the temporal bone and the lack of a facial nerve, since in this simulator the nerve was only painted there. Despite these criticisms, which will serve to enhance the simulator, dissection was deemed satisfactory by the surgeons who evaluated it.

## CONCLUSION

Dissection in this otologic simulator does not replace the actual dissection of temporal bones from cadavers; nonetheless, it is a promising alternative for the practical teaching of otologic surgery. With the growing difficulty in obtaining natural temporal bones, we must develop new teaching tools for the continuous improvement of surgeons.
